# New variant in *PLP1* gene associated with X-linked spastic paraplegia type 2: First report of a family in Colombia

**DOI:** 10.7705/biomedica.7733

**Published:** 2026-06-01

**Authors:** Nicolás Laverde-Sudupe, Ángela Lizeth Giraldo-Serna, Diana Ramírez-Montaño, Julián Ramírez-Cheyne, Wilmar Saldarriaga-Gil

**Affiliations:** 1 Facultad de Ciencias de la Salud, Departamento de Medicina, Pontificia Universidad Javeriana, Cali, Colombia Pontificia Universidad Javeriana Facultad de Ciencias de la Salud Departamento de Medicina Pontificia Universidad Javeriana Cali, Colombia; 2 Departamento de Morfología, Escuela de Ciencias Básicas, Facultad de Salud, Universidad del Valle, Cali, Colombia Universidad del Valle Departamento de Morfología Escuela de Ciencias Básicas, Facultad de Salud Universidad del Valle Cali, Colombia; 3 Unidad de Medicina Genómica y Genética, Clínica Imbanaco, Cali, Colombia Unidad de Medicina Genómica y Genética Clínica Imbanaco Cali, Colombia

**Keywords:** Spastic paraplegia, hereditary, Pelizaeus-Merzbacher disease, paraplejia espástica hereditaria, enfermedad de Pelizaeus-Merzbacher

## Abstract

Hereditary spastic paraplegias are genetic disorders characterized by spasticity in the lower limbs, weakness, and sensory disturbances. Global prevalence ranges from 1.27 to 9.6 per 100 000 individuals. Mutations in the *PLP1* gene cause various X-linked hereditary spastic paraplegias phenotypes, including Pelizaeus-Merzbacher disease and spastic paraplegia type 2.

A 53-year-old male presented with chronic lower limb weakness since childhood, requiring a wheelchair at age 47 and exhibiting zero strength in the lower limbs. Magnetic resonance imaging revealed periventricular leukodystrophy; similar symptoms were found in maternal uncles and a nephew. The 32-year-old nephew had gait difficulties. A genetic sequencing panel identified a hemizygous variant of uncertain significance in the *PLP1* gene [c.197A>T (p.His66Leu)] in both, not reported in population genetic databases.

X-linked spastic paraplegia 2 is a disease primarily affecting gait and causing lower limb weakness. Reports indicate that it may also include cognitive impairment, nystagmus, and ataxia, although in the studied family, weakness predominated without cerebellar symptoms. Thirty-six families with this condition have been documented worldwide, with cases of asymptomatic carrier females. The c.197A>T (p.His66Leu) variant in the *PLP1* gene, identified in this case, is novel and, despite being classified as “of uncertain significance”, could be pathogenic according to bioinformatic predictors, explaining the spastic paraplegia 2 presentation in this family.

## Resumen

Las paraplejias espásticas hereditarias son trastornos genéticos que se caracterizan por espasticidad de los miembros inferiores, debilidad y alteración de la sensibilidad. Su prevalencia global varía entre 1,27 y 9,6 por cada 100 000 personas. Las mutaciones del gen que codifica para la proteína proteolipídica provocan diferentes fenotipos de paraplejias espásticas hereditarias ligadas a X, incluyendo la enfermedad de Pelizaeus y la paraplejia espástica hereditaria de tipo 2.

Se presenta el caso de un paciente masculino de 53 años con debilidad crónica de los miembros inferiores desde la infancia, requirió silla de ruedas a los 47 años y tenía fuerza nula de los miembros inferiores. En la resonancia magnética se observó leucoencefalopatía periventricular. Se encontraron síntomas similares en los tíos maternos y en un sobrino. El sobrino, de 32 años, tenía dificultades en la marcha. Un panel genético de secuenciación identificó una variante hemicigota de significado clínico incierto en el gen *PLP1* (c.197A>T (p.His66Leu)) tanto en el paciente como en el sobrino, no reportada en las bases genéticas de datos poblacionales.

La paraplejia espástica hereditaria de tipo 2, ligada a X, es una enfermedad que causa debilidad de los miembros inferiores. Los reportes señalan que esta enfermedad también puede incluir alteración cognitiva, nistagmo y ataxia, aunque en la familia estudiada predominó la debilidad sin síntomas cerebelosos.

A nivel mundial, se han documentado 36 familias con esta condición, con casos de mujeres portadoras asintomáticas. La mutación c.197A>T (p.His66Leu) del gen *PLP1*, identificada en este caso, es nueva y, aunque está clasificada como «de significado incierto», podría ser patogénica -según los predictores bioinformáticos-, explicando la presentación de la paraplejia espástica hereditaria de tipo 2 en esta familia.

Hereditary spastic paraplegias constitute a group of genetic disorders sharing common characteristics such as spasticity in the lower limbs, weakness, hyperreflexia, and impaired vibration sense. The estimated prevalence worldwide ranges from 1.27 to 9.6 per 100 000 individuals depending on the specific paraplegia ^1^. Hereditary spastic paraplegias have been classified into early-onset when symptoms begin in early childhood and can be non-progressive or late-onset starting in late childhood or adulthood and progressing over time.

Additionally, hereditary spastic paraplegias have been categorized into uncomplicated and complicated forms. The former is characterized by neurological alterations limited to weakness and progressive spasticity in the lower limbs, and urinary disturbances. The latter encompasses the symptoms of the uncomplicated form, but includes more extensive neurological involvement with ataxia, nystagmus, seizures, dementia, muscle atrophy, among others ^2^. Within this spectrum, hereditary spastic paraplegias with autosomal dominant, autosomal recessive, X-linked, and mitochondrial inheritance are identified, with the latter being the rarest form, affecting only 1-2% of individuals.

Pathogenic variants in the *PLP1* gene give rise to different types of diseases. These conditions are characterized by a phenotypic spectrum with two extremes: at one end, Pelizaeus-Merzbacher disease (MIM 312080), which typically manifests between the neonatal period and five years of age with nystagmus, hypotonia, severe spasticity, ataxia, seizures, and cognitive impairment. At the other end, X-linked hereditary spastic paraplegia 2 (MIM 312920, SPG2) is characterized by progressive and limiting spasticity in the lower limbs associated with hyperreflexia, spastic gait, contractures, and pes cavus ^3^. It is caused by pathogenic variants in the *PLP1* gene located at *locus* Xq22, which encodes the proteolipid protein *PLP1* and a splice variant DM20, representing more than 50% of the total mass of myelin-associated proteins in the central nervous system ^4^. To date, 32 pathogenic variants causing spastic paraplegia 2 have been reported, including 12 missense, seven frameshift, four splicing, six intronic, and three nonsense variants ^5^.

We present a novel variant of uncertain significance in the *PLP1* gene associated with the phenotype of spastic paraplegia 2, being the first reported case in a Colombian family.

## Case presentation

**Figure 1.**
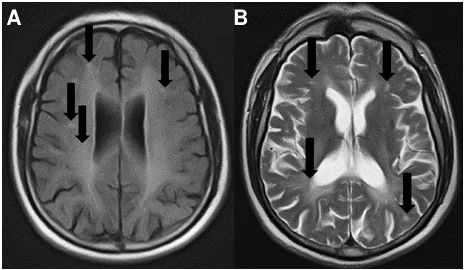
A and B show the brain magnetic resonance imaging of patient 1. Image A demonstrates periventricular predominant leukodystrophy on a FLAIR sequence, the optimal sequence for assessing white matter abnormalities. Image B reveals hyperintense findings consistent with leukodystrophy, primarily in the parieto-occipital region.

**Figure 2.**
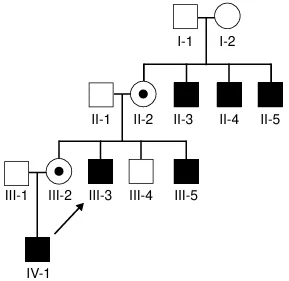
Family pedigree demonstrating an X-linked inheritance pattern, with affected males and carrier females

A 53-year-old man presented to the genetics clinic for follow-up due to chronic weakness in the lower limbs since childhood. He experienced multiple falls before the age of 40, with impaired gait and requiring assistance with walking. At the age of 47, he required a wheelchair and, at the time of evaluation, he was unable to walk or stand. Physical examination revealed 4/5 strength in the upper limbs, 0/5 strength in the lower limbs, generalized spasticity, bilateral hyperreflexia, with no positive Babinski or Hoffman signs, and increased muscle tone. Brain magnetic resonance imaging (MR) performed at the age of 52 showed periventricular leukodystrophy without contrast enhancement (figure 1). Family history revealed a similar neurological phenotype in maternal uncles and a nephew of his sister, with no documented involvement in women (figure 2).

The 32-year-old nephew was also evaluated in the genetics clinic, reporting a clinical picture that began at the age of 21, characterized by progressive difficulty and instability with walking without requiring support until now. Nerve conduction studies were performed in all four limbs at the age of 32, along with cervical and spinal MR, which were normal. No findings were found to explain the condition.

Given the suspicion of a hereditary condition, a next-generation sequencing (NGS) genetic panel for genes related to hereditary spastic paraplegias was performed in the 53-year-old patient, finding a hemizygous variant of uncertain significance in the *PLP1* gene (NM_000533.5):c.197A>T p.(His66Leu), which was confirmed by Sanger sequencing in the 32-year old nephew. This variant has not been reported in the ClinVar database, The Human Gene Mutation Database (HGMD), or GenomeAD, and, according to the American College of Medical Genetics and Genomics (ACMG) criteria, it is classified as a variant “of uncertain significance”.

### Ethical considerations

This manuscript corresponds to a case report, which is classified under Resolution 8430 of 1993 as research without ethical risk. Informed consent was obtained from the patient and family members. All information was ensured to remain anonymous.

## Discussion

X-linked spastic paraplegia 2 is a disorder characterized by a central nervous system alteration, primarily affecting gait, secondary to lower limb weakness, generating symptoms of upper motor neuron disease with the presence of hyperreflexia and a positive Babinski sign. Yao *et al*. ^5^ described in 2023 three families in which the most common neurological manifestation after the characteristic weakness was cognitive impairment, followed by nystagmus, dysarthria, ataxia, and urinary incontinence. In this family, the primary symptom described was lower limb weakness without ataxic or cerebellar signs, and without evidence of altered higher functions (table 1). This may be due to the impact of the genetic variant on protein function, which could reflect a possibly slower progression, leading to a delay in manifestations at older ages.

**Table 1.** Classic phenotype of patients with spastic paraplegia 2 is compared to that of the family studied in this research

Classic phenotype	Presentation of the disease in the family studied
X-linked recessive	Yes
Nystagmus	No
Optic atrophy	No
Joint contractures	No
Pes cavus	No
Lower limb weakness	Yes
Lower limb spasticity	Yes
Spastic gait	Yes
Hyperreflexia	Yes
Plantar extensor response	No
Dysarthria	No
Dysmetria	No
Ataxia	No
Cerebellar signs	No
Mental retardation	No
Upper limb involvement	No
Degeneration of lateral corticospinal tracts	No
Degeneration of spinocerebellar tracts	No
Childhood onset	No
Caused by mutation in *PLP1* gene	Yes

Worldwide, a total of 36 families with this condition have been reported, with 66 affected males and 16 females. Although the inheritance of the disease corresponds to an X-linked recessive mechanism, and presentation in female subjects would be limited to homozygous patients, it has been described that the phenomenon of random X chromosome inactivation can lead to milder manifestations in female patients, a phenomenon described in other conditions with this inheritance mechanism such as Duchenne muscular dystrophy ^6^ and hemophilia ^7^. Yamamoto-Shimojina *et al*. ^8^ reported a case of a female patient with a heterozygous variant in the *PLP1* gene with late-onset gait and balance disturbances. In the presented family, female individuals to date have a normal neurological examination, behaving as asymptomatic carriers of this condition.

Kubota *et al*. ^9^ described that in MR, patients with spastic paraplegia 2 present abnormalities described as “patches” in the T2 sequence or diffuse leukoencephalopathy, primarily affecting the periventricular, parieto-occipital, internal capsule, corpus callosum, and cerebellar peduncle regions. Patient 1 presented with periventricular leukodystrophy, which correlates directly with the literature (figure 2). On the other hand, in the neuroimaging of subject 2, no conclusive findings were reported in the MR. This can be explained by a gradual progression of the disease, which would lead to a progression in the findings of central nervous system images correlating with clinical progression.

The type of variant identified in the *PLP1* gene correlates with the phenotypic presentation of the spectrum of this disease. The most severe presentation, known as Pelizaeus-Merzbacher syndrome, is generally caused by copy number variations such as duplications of significant segments within the gene. Specifically, if the involvement includes exon 3, which is a critical point for alternative splicing of the protein, an altered splice can be found that allows the generation of the transcript encoding the DM20 protein ^10^. Likewise, in some patients with the Pelizaeus-Merzbacher phenotype, it is due to missense alterations (especially the substitution of conserved amino acids) at critical points of the protein ^10^. Moreover, the presentation of spastic paraplegia 2 is caused by variants with changes in more conserved residues and in less critical regions of the protein ^11^, leading to milder phenotypes and less central nervous system involvement compared to the Pelizaeus-Merzbacher phenotype.

The variant presented here generates a change from an adenine to a thymine in exon 3, creating a change in the protein that leads to the substitution of the histidine amino acid for a leucine, a moderately conserved amino acid evolutionary. This results in an accumulation of a misfolded proteolipid protein (PLP) in the endoplasmic reticulum with toxic overexpression and loss of its function ^12^. The PLP protein plays a very important role in the stabilization and maintenance of myelin sheets and the development of oligodendrocyte precursors and glial cells ^13^. The expression of a non-functional protein directly leads to the fragility of these multilamellar myelin sheets and makes these neurons susceptible to demyelination, presenting the different phenotypes already mentioned ^14^.

In this report, a missense mutation c.197A>T (p.His66Leu) was identified that had not been previously reported in the 1000 Genomes Project, ExAC, gnomAD, or ClinVar, and is therefore considered a novel variant (table 2). Based on the American College of Medical Genetics and Genomics (ACMG) variant interpretation guidelines, this variant is classified as a variant “of uncertain significance” ^15^.

**Table 2.** Prediction of novel variant *PLP1*

Location on DNA		
	Genomic position	chrX 103786470
	Codon change	cAt/cTt
	Position in DNA	c.197A>T
	Position in protein	p.(His66Leu)
Effect predictions (variant predictor tool)		
	LRT, DANN, mutation tester	Benign supporting (score = 0.001664)
	SIFT	Uncertain (score = 0.011)
	PROVEAN	Uncertain (score = -3.76)
	DEOGEN2	Pathogenic moderate (score = 0.9439)
	MutPred	Pathogenic supporting (score = 0.639)
	FATHMM	Deleterious moderate (score = -5.53)
	BayesDel	Deleterious supporting (score = 0.2)
Occurrence in public databases		
	dbSNP v.150	Not present
	1,000 Genome Project	Not present
	ExAC	Not present
	gnomAD	Not present
	Classification of variant	Conflicted: likely pathogenic and variant of uncertain significance
	ACMG guideline for the interpretation of sequence variant	1 moderate criteria: PM2; and two supporting criteria PP2-PP3

ACMG: American College of Medical Genetics and Genomics

Based on the clinical findings observed in this family and the current understanding of PLP1-related spastic paraplegia 2, we propose a practical clinical management protocol aimed at guiding the diagnostic approach, follow-up, and genetic evaluation of affected individuals and at-risk relatives. This protocol summarizes key recommendations for neurological monitoring, neuroimaging, genetic testing, counseling, and multidisciplinary care, providing a structured framework that may support clinicians in standardizing the evaluation and long-term management of patients with X-linked hereditary spastic paraplegia. The proposed steps are outlined in table 3.

**Table 3.** Proposed clinical management protocol for patients with spastic paraplegia type 2 associated with *PLP1* variants

Management area	Clinical recommendations
Neurological evaluation	Comprehensive neurological assessment every 6-12 months Systematic evaluation of strength, tone, spasticity, reflexes, gait, and functional capacity
Neuroimaging	Baseline brain MR with FLAIR sequences to characterize leukodystrophy Repeat MR based on clinical progression or emergence of new symptoms
Electrophysiology	Perform only if diagnostic uncertainty exists, as nerve conduction studies are typically normal in spastic paraplegia type 2
Genetic testing	Order a targeted hereditary spastic paraplegia gene panel when the phenotype suggests X-linked inheritance Confirm identified variants via Sanger sequencing in affected relatives
Genetic counseling	Explain X-linked recessive inheritance pattern Identify and counsel female carriers Discuss reproductive risks and options for prenatal or preimplantation diagnosis
Rehabilitation and functional management	Physical therapy focused on spasticity control, stretching, upper-limb strengthening, and gait training when feasible Assess the need for orthotic devices or mobility aids according to disease progression
Symptomatic and multidisciplinary care	Urological evaluation for urinary symptoms Neuropsychological assessment when cognitive involvement is suspected Include physiatry, rehabilitation, and social work as part of longitudinal care
Family screening and follow-up	Periodic monitoring of at-risk male relatives Evaluate female carriers to detect subtle or late-onset manifestations

FLAIR: Fluid-Attenuated Inversion Recovery; MR: Magnetic resonance imaging

## Conclusion

This article reports the first Colombian family with spastic paraplegia 2, considered a rare condition, as well as a novel variant in the *PLP1* gene absent from worldwide population databases. Pathogenic variants in the *PLP1* gene generate a disease spectrum that varies from the most severe presentation, corresponding to the Pelizaeus-Merzbacher phenotype, to milder forms that correspond to spastic paraplegia 2, depending on the type of genetic variant, its effect on the splicing process, and the derived transcripts, as well as protein function.

This novel, unpublished variant, is associated with a severe phenotype within the variable expressivity of the disease. Interdisciplinary followup should continue in the members of the reported family to establish a genotype-phenotype correlation in this type of disease.

By reporting such cases, rare conditions become more visible, increasing the ability to suspect them based on the patient’s clinical presentation, but particularly when there is a family history suggesting distinguishable inheritance patterns such as X-linked recessive inheritance.
